# Supplementary material for: Modeling spatial and temporal variability in educational development index of Bangladesh using socio-economic, and demographic data, 2001–2021: A Bayesian approach
Source: PLoS One. 2025 Mar 28;20(3):e0317030. doi: 10.1371/journal.pone.0317030 (PMC11952229; doi:10.1371/journal.pone.0317030)
Supplement: Attachment — Submitted filename: PONE-D-24-14527_with comments.pdf [file pone.0317030.s002.pdf]

PLOS ONE

Modeling Temporal and Spatial Variability in Educational Development Index of Bangladesh: A Bayesian Approach

--Manuscript Draft--

**Manuscript Number:**	PONE-D-24-14527
**Article Type:**	Research Article
**Full Title:**	Modeling Temporal and Spatial Variability in Educational Development Index of Bangladesh: A Bayesian Approach
**Short Title:**	Modeling Temporal and Spatial Variability in Educational Development Index of Bangladesh
**Corresponding Author:**	Md. Moyazzem Hossain<br>Jahangirnagar University<br>Savar, Dhaka BANGLADESH
**Keywords:**	Bayesian Inference; Educational Development Index; MCMC; Spatial-Temporal Approach
**Abstract:**	Background: The Educational Development Index (EDI) is a critical tool for assessing and tracking the progress of education systems from local to national, and even global scales and needs to be chosen for every layer of the subnational boundaries to secure the basic human rights of the people. In reality, there are significant variations within the consecutive time breaks and the geographical boundaries that need to be examined. The authors aim to examine how the educational development index relates to various spatiotemporal variables.<br>Methods and Materials: This research is based on secondary data on literacy rates from 64 districts of Bangladesh and 6 relevant variables over the period 2001 to 2021. The optimal model for the data was identified from Bayesian spatial-temporal modeling (Linear, ANOVA, AR1, and AR2) and the Markov Chain Monte Carlo (MCMC) method used to generate data about the prior and posterior realizations. To select the best model different model selection and validation criteria (Deviance Information Criterion (DIC), Watanabe-Akaike information criterion (WAIC), and Route Means Square Error (RMSE)) were employed in this study.<br>Results: Among spatiotemporal models, the AR (1) model showed better performance compared with others. Significant spatial (=0.994) and temporal (=0.347) variations were identified for the suited model. However, the health index, income index, expected years of schooling, population density, and dependency ratio are important components of educational development in Bangladesh.<br>Conclusion: The variation in the spatial domain can be used to identify the districts to improve the educational index controlling responsible factors by the policymakers.
**Order of Authors:**	Afroza Sultana
	Md. Akher Ali
	Md. Sifat Ar Salan
	Mohammad Alamgir Kabir
	Md. Moyazzem Hossain
**Opposed Reviewers:**	
**Additional Information:**	
**Question**	**Response**
**Financial Disclosure**<br><br>Enter a financial disclosure statement that describes the sources of funding for the work included in this submission. Review the submission guidelines for detailed	The author(s) received no specific funding for this work.

Powered by Editorial Manager® and ProduXion Manager® from Aries Systems Corporation

requirements. View published research articles from *PLOS ONE* for specific examples.<br><br>This statement is required for submission and **will appear in the published article** if the submission is accepted. Please make sure it is accurate.<br><br>**Funded studies**<br>Enter a statement with the following details:<br>• Initials of the authors who received each award<br>• Grant numbers awarded to each author<br>• The full name of each funder<br>• URL of each funder website<br>• Did the sponsors or funders play any role in the study design, data collection and analysis, decision to publish, or preparation of the manuscript?<br><br>Did you receive funding for this work?	
**Competing Interests**<br><br>Use the instructions below to enter a competing interest statement for this submission. On behalf of all authors, disclose any competing interests that could be perceived to bias this work—acknowledging all financial support and any other relevant financial or non-financial competing interests.<br><br>This statement is required for submission and **will appear in the published article** if the submission is accepted. Please make sure it is accurate and that any funding sources listed in your Funding Information later in the submission form are also declared in your Financial Disclosure statement.<br><br>View published research articles from *PLOS ONE* for specific examples.	The authors have declared that no competing interests exist.

**NO authors have competing interests**<br><br>Enter: *The authors have declared that no competing interests exist.*<br><br>**Authors with competing interests**<br><br>Enter competing interest details beginning with this statement:<br><br>*I have read the journal's policy and the authors of this manuscript have the following competing interests: [insert competing interests here]*<br><br>* typeset	
**Ethics Statement**<br><br>Enter an ethics statement for this submission. This statement is required if the study involved:<br><br>• Human participants<br>• Human specimens or tissue<br>• Vertebrate animals or cephalopods<br>• Vertebrate embryos or tissues<br>• Field research<br><br>Write "N/A" if the submission does not require an ethics statement.<br><br>General guidance is provided below. Consult the submission guidelines for detailed instructions. **Make sure that all information entered here is included in the Methods section of the manuscript.**	N/A

**Format for specific study types**<br><br>**Human Subject Research (involving human participants and/or tissue)**<br>• Give the name of the institutional review board or ethics committee that approved the study<br>• Include the approval number and/or a statement indicating approval of this research<br>• Indicate the form of consent obtained (written/oral) or the reason that consent was not obtained (e.g. the data were analyzed anonymously)<br><br>**Animal Research (involving vertebrate animals, embryos or tissues)**<br>• Provide the name of the Institutional Animal Care and Use Committee (IACUC) or other relevant ethics board that reviewed the study protocol, and indicate whether they approved this research or granted a formal waiver of ethical approval<br>• Include an approval number if one was obtained<br>• If the study involved *non-human primates*, add *additional details* about animal welfare and steps taken to ameliorate suffering<br>• If anesthesia, euthanasia, or any kind of animal sacrifice is part of the study, include briefly which substances and/or methods were applied<br><br>**Field Research**<br><br>Include the following details if this study involves the collection of plant, animal, or other materials from a natural setting:<br>• Field permit number<br>• Name of the institution or relevant body that granted permission	
**Data Availability**<br><br>Authors are required to make all data underlying the findings described fully available, without restriction, and from the time of publication. PLOS allows rare exceptions to address legal and ethical concerns. See the PLOS Data Policy and FAQ for detailed information.	Yes - all data are fully available without restriction

Powered by Editorial Manager® and ProduXion Manager® from Aries Systems Corporation

A Data Availability Statement describing where the data can be found is required at submission. Your answers to this question constitute the Data Availability Statement and **will be published in the article**, if accepted.<br><br>**Important:** Stating 'data available on request from the author' is not sufficient. If your data are only available upon request, select 'No' for the first question and explain your exceptional situation in the text box.<br><br>Do the authors confirm that all data underlying the findings described in their manuscript are fully available without restriction?	
**Describe where the data may be found in full sentences. If you are copying our sample text, replace any instances of XXX with the appropriate details.**<br><br>• If the data are **held or will be held in a public repository**, include URLs, accession numbers or DOIs. If this information will only be available after acceptance, indicate this by ticking the box below. For example: *All XXX files are available from the XXX database (accession number(s) XXX, XXX.*).<br>• If the data are all contained **within the manuscript and/or Supporting Information files**, enter the following: *All relevant data are within the manuscript and its Supporting Information files.*<br>• If neither of these applies but you are able to provide **details of access elsewhere**, with or without limitations, please do so. For example:<br><br>*Data cannot be shared publicly because of [XXX]. Data are available from the XXX Institutional Data Access / Ethics Committee (contact via XXX) for researchers who meet the criteria for access to confidential data.*<br><br>*The data underlying the results presented in the study are available from (include the name of the third party*	All Data are available in the website of Global Data Lab (https://www.globaldatalab.org) and Bangladesh Bureau of Statistics (https://bbs.gov.bd/).

and contact information or URL).<br>• This text is appropriate if the data are owned by a third party and authors do not have permission to share the data.<br><br>* typeset	
Additional data availability information:	

Powered by Editorial Manager® and ProduXion Manager® from Aries Systems Corporation

April 10, 2024

Dear
Editor-in-Chief

Subject: Submission of Manuscript for Evaluation

Dear Editor,
I am enclosing herewith the manuscript entitled "***Modeling Temporal and Spatial Variability in Educational Development Index of Bangladesh: A Bayesian Approach***" submitted to your journal. The Educational Development Index (EDI) is a critical tool for assessing and tracking the progress of education systems from local to national, and even global scales and needs to be chosen for every layer of the subnational boundaries to secure the basic human rights of the people. In reality, there are significant variations within the consecutive time breaks and the geographical boundaries that need to be examined. The authors aim to examine how the educational development index relates to various spatiotemporal variables.

With the submission of this manuscript, I would like to undertake that the above-mentioned manuscript has not been published elsewhere, accepted for publication elsewhere, or under editorial review for publication elsewhere. It is an original article. The authors declare no conflicts of interest. The final version is approved by all authors for publication. I would appreciate it if you could consider our manuscript for publication in your journal.

Sincerely Yours,

Md. Moyazzem Hossain

Professor,
Department of Statistics and Data Science,
Jahangirnagar University,
Savar, Dhaka-1342, Bangladesh
Email: hossainmm@juniv.edu
ORCiD: https://orcid.org/0000-0003-3593-6936

Manuscript
Click here to access/download;Manuscript;Manuscript_Educational

Modeling Temporal and Spatial Variability in Educational Development Index of Bangladesh: A Bayesian Approach

Afroza Sultana[1,*], Md. Akher Ali[1], Md. Sifat Ar Salan[1], Mohammad Alamgir Kabir[1], Md. Moyazzem Hossain[1,*]

[1]Department of Statistics and Data Science, Jahangirnagar University, Savar, Dhaka-1342, Bangladesh, Email: afrozasultana5161@gmail.com (AS), akherali4455@gmail.com (MAA), sifat@juniv.edu (SAS), alamgir@juniv.edu (MAK), hossainmm@juniv.edu (MMH)

*Corresponding author

Abstract

Background: The Educational Development Index (EDI) is a critical tool for assessing and tracking the progress of education systems from local to national, and even global scales and needs to be chosen for every layer of the subnational boundaries to secure the basic human rights of the people. In reality, there are significant variations within the consecutive time breaks and the geographical boundaries that need to be examined. The authors aim to examine how the educational development index relates to various spatiotemporal variables.

Methods and Materials: This research is based on secondary data on literacy rates from 64 districts of Bangladesh and 6 relevant variables over the period 2001 to 2021. The optimal model for the data was identified from Bayesian spatial-temporal modeling (Linear, ANOVA, AR1, and AR2) and the Markov Chain Monte Carlo (MCMC) method used to generate data about the prior and posterior realizations. To select the best model different model selection and validation criteria (Deviance Information Criterion (DIC), Watanabe-Akaike information criterion (WAIC), and Route Means Square Error (RMSE)) were employed in this study.

Results: Among spatiotemporal models, the AR (1) model showed better performance compared with others. Significant spatial (ρ_S=0.994) and temporal (ρ_T=0.347) variations were identified for

the suited model. However, the health index, income index, expected years of schooling, population density, and dependency ratio are important components of educational development in Bangladesh.

Conclusion: The variation in the spatial domain can be used to identify the districts to improve the educational index controlling responsible factors by the policymakers.

Keywords: Bayesian Inference; Educational Development Index; MCMC; Spatial-Temporal Approach

Introduction

Education is a fundamental pillar of economic and social development, playing a vital role in shaping the sustainable and digitalized future of nations. It provides insights into the country's progress and well-being in various dimensions, including economic, social, health, and environmental factors [1]. Education fosters the development of human skills, which are viewed as human capital since they enable the community to access the services it requires [2]. Better educational opportunities combined with a trained workforce indicate better opportunities for economic growth and development in general. According to international and regional human rights law and other international texts, such as the Universal Declaration of Human Rights [3] education is acknowledged as one of the essential human rights [4]. Increased labor market involvement, better child and family health, improved nutrition, a reduction in poverty, and increased life possibilities are all benefits of education The Educational Development Index (EDI) serves as an overall measure that includes a variety of educational indicators, reflecting the overall quality and accessibility of education within a particular region or country. In the context of Bangladesh, a developing country striving for progress with an area of 147,570 square kilometers and a density of 1,156.84 per square kilometer [5], understanding temporal and spatial variations in the ~~educational development index (~~EDI~~)~~ is crucial. Analyzing the spatiotemporal distribution of EDI in Bangladesh can provide insight into the performance of the educational system, identify areas for improvement, and suggest the allocation of resources to ensure there are relationships across sectors. Bangladesh's remarkable development journey has turned it from a nation affected

by war into a growing and promising economy, often referred to as the "Bangladesh Miracle" [6]. Bangladesh has increased efforts to improve the quality of education because it recognizes that education is essential to achieving the desired degree of national development. The nation is committed to achieving Sustainable Development Goal (SDG) 4, which focuses on ensuring inclusive, equitable, and high-quality education while promoting lifelong learning opportunities for everyone, along with the associated targets. The nation has successfully achieved gender parity in alignment with the Millennium Development Goals. [4]. In Bangladesh, the government, non-governmental organizations (NGOs), and international agencies have been working tirelessly to put these words into action by improving the quality of education. Bangladeshi policymakers believe that with quality education, our population may be transformed into a useful resource for national development [7]. Despite constitutional obligations for balanced growth, regional disparities persist in Bangladesh. However, the government of our nation must address the idea of balanced regional growth because it is required under the constitution [8].

In addition to being a fundamental human right and a means of reducing poverty, education also fosters social equality and economic growth by producing skilled workers. [9] support the use of geodemographics in assessing potential education inequality by utilizing a spatial approach that is demonstrated in a case study of central Beijing. A community's educational attainment is a key component of its social and economic development and a predictor of future progress [10]. However, the quality, equity, and appropriateness of accessibility as well as the spatial distribution of the facilities produce differences in the efficiency of education which is proved from the write of Bulti [11]. Regional variations exist in the literacy rate, expected years of schooling, health index, income index, child health, life expectancy, etc. Spatial data mining, a critical facet of Exploratory Spatial Data Analysis (ESDA), gains significance with modern computing power and abundant geospatial data. This study applies ESDA techniques to Bangladesh's geospatial data, exploring literacy rates and educational establishment distribution in light of Tobler's first law of geography [12]. This research endeavors to delve into the temporal and spatial variance in the EDI of Bangladesh using a Bayesian framework approach which offers a powerful analytical tool for modeling complex systems, incorporating uncertainty, and making robust predictions based on data.

In this research we have investigated spatial patterns and autocorrelation to identify the most suitable spatial model, potentially aiding decision-makers [13]. ~~The hierarchical Bayesian autoregressive model a proposal put out by Sahu is the spatiotemporal model used to model educational development data [14].~~ The Bayesian Hierarchical Spatial-temporal model, rooted in the Bayesian framework, is employed to analyze the fluctuations within spatial regions and their temporal evolution. This model effectively integrates both spatial and temporal dimensions, making it a valuable tool for modeling social data [15], given the significant variability in development across different periods and geographical locations. Therefore, we hope that the findings will offer insightful information to scholars examining the influence of different social factors on global educational growth.

Materials and Method

Data Source

The Global Data Lab's Area Database (https://www.globaldatalab.org) was considered the main source of data that is accessed and free to use [16]. The GDL is an independent data and research center at the Nijmegen School of Management (https://www.ru.nl/nsm/) of Radboud University (https://www.ru.nl/). Bangladesh Bureau of Statistics (BBS) is also used as a secondary source for our data [5]. The data was collected for the time duration of 2001 to 2021. The second level of administrative was considered as the spatial domain of this study, this is why all the data was collected for each district and each predefined period (yearly).

Study Variables

The educational development index is a composite measure of educational outcomes in different regions of Bangladesh which was the main response variable of this study. In this research, we have considered the percentage of literacy rate according to the district for the predefined period as our response variable. Based on some previous studies we have considered income index, health index, and expected years of schooling as our covariates to justify our objectives (S. Ahmed, 2014; Khan, 2010; Sarma et al., 2017; Street, 1995; Vanderslott et al., 2013). Population density, distance

from Dhaka (the capital of Bangladesh), and dependency ratio are also used as covariates for our study which were found in a similar study (M. Ahmed & Rahamāna, 2013; Kabir & Akter, 2014; Mazumdar, 2005). In addition, there were 64 different district names in the spatial dimension, and the temporal dimension covered 21 years in a row in these districts. The aggregate data for the 64 districts throughout the 21 years is represented by the 1344 observations (rows) each variable contains.

Spatio-Temporal Modeling

Studies that document and evaluate both the locations and times of the observations are referred to as spatio-temporal modeling. The main focus of spatio-temporal analysis was on variation in the variable in combinations of place and time units over the study area and study period. Different types of separable and non-separable models were employed to observe variation in spatiotemporal datasets.

Assumption Checking

An important assumption of spatio-temporal modeling is the exitance of spatio-temporal autocorrelation. Here, autocorrelation in spatio-temporal data has been analyzed using Moran's I (Global and Local Moran's *I*) and Geary's C [24]. Although in theory Geary's coefficient and Moran's index are comparable, in actuality Geary's coefficient is based on a sample and Moran's index is based on the population [25,26]. In his study, spatio-temporal autocorrelation checking has been done as an extension of Moran's I and Geary's C [27].

Bayesian Hierarchical Model

Here, the response variable literacy rate $\boldsymbol{Y_{it}}$ for i = 1, 2, ..., 64 and t = 1, 2, ..., 21 was assumed to follow the Gaussian distribution $\boldsymbol{Y_{it}} \sim \mathbf{N}\ (\boldsymbol{\mu_{it}}, \boldsymbol{\sigma^2}_{it})$. And probability mass function was denoted as,

$$(\boldsymbol{y_{it}}, \boldsymbol{\mu_{it}}, \boldsymbol{\sigma^2}_{it}) = \frac{1}{\sqrt{2\pi\sigma^2_{it}}}\ \boldsymbol{e}^{\left(-\frac{1}{2\sigma^2_{it}}(y_{it}-\mu_{it})^2\right)}\ , \boldsymbol{\mu_{it}} \epsilon \mathbb{R},\ \boldsymbol{y_{it}} \epsilon \mathbb{R}$$

Where, $\boldsymbol{y_{it}}$ is literacy rate, $\boldsymbol{\mu_{it}}$ is the mean and $\boldsymbol{\sigma^2}_{it}$ is the variance of the defined response variable at space-time combination. Let's say that $\boldsymbol{x_{it}}$ represents the covariates of p-dimensional at the space-time combination. The model can be written as

$$\boldsymbol{Y_{it}} \sim \boldsymbol{x'_{it}\beta} + \boldsymbol{\psi_{it}}$$

For independent error general linear regression model $\boldsymbol{\beta}$ is a matrix of unknown regression coefficient follows and $\boldsymbol{\psi_{it}}$ is the error term that we assume to follows $N(0, \boldsymbol{\sigma^2})$. Based on different assumptions about their construction, the random $\boldsymbol{\psi_{it}}$ is considered to have the following varied range of models. To extend the process and parameter models to both space and time dimensions, several models have been used in research [14]and Knorr-Held proposed an analysis of the variance type model with or without the inclusion of the space-time interaction (Knorr-Held & Raber, 2000). The general spatial-temporal models were considered as,

$$\boldsymbol{\psi_{it}} = \begin{cases} \boldsymbol{\beta_1} + \boldsymbol{\phi_i} + (\boldsymbol{\beta_2} + \boldsymbol{\delta_i})\dfrac{\boldsymbol{t} - \bar{\boldsymbol{t}}}{\boldsymbol{T}}, & \text{Linear model of trend} \\ \boldsymbol{\phi_i} + \boldsymbol{\delta_i} + \boldsymbol{\gamma_{it}}, & \text{ANOVA model} \\ \boldsymbol{\phi_{it}} + \boldsymbol{\delta_t}, & \text{AutoRegressive model} \end{cases}$$

In this case, the overall intercept and the slope (trend) parameters, denoted as $\boldsymbol{\beta_1}$ and $\boldsymbol{\beta_2}$, respectively, in the Bayesian model have a flat prior distribution. The incremental intercept and slope parameters unique to the $\boldsymbol{i^{th}}$ area is indicated by the parameters $\boldsymbol{\phi_i}$ and $\boldsymbol{\delta_i}$. A modified Conditional Autoregressive (CAR) prior distribution is applied to both sets of parameters, and different values of $\boldsymbol{\rho}$ and $\boldsymbol{\tau^2}$are used. The parameters $\boldsymbol{\phi} = \boldsymbol{\phi_{1\ldots,}}\boldsymbol{\phi_{64}}$ and $\boldsymbol{\delta} = \boldsymbol{\delta_{1\ldots,}}\boldsymbol{\delta_{64}}$ are assigned the **NCAR** ($\boldsymbol{\phi}|\boldsymbol{\rho_{int}}, \boldsymbol{\tau^2}_{\boldsymbol{int}}, \boldsymbol{W}$) and **NCAR** ($\boldsymbol{\delta}|\boldsymbol{\rho_{slo}}, \boldsymbol{\tau^2}_{\boldsymbol{slo}}, \boldsymbol{W}$) distribution. Here $\boldsymbol{\rho_{int}}$, $\boldsymbol{\rho_{slo}}$, $\boldsymbol{\tau^2}_{\boldsymbol{slo}}$, $\boldsymbol{\tau^2}_{\boldsymbol{slo}}$ are autoregression and variance parameters for the intercept ($\boldsymbol{\phi_i}$) and slope ($\boldsymbol{\delta_i}$) processes.

The variance parameters, $\boldsymbol{\tau^2}_{\boldsymbol{int}}$ and $\boldsymbol{\tau^2}_{\boldsymbol{slo}}$, follow inverse gamma prior distributions, whilst the parameters $\boldsymbol{\rho_{int}}$ and $\boldsymbol{\rho_{slo}}$ are assigned independent uniform prior distributions inside the unit interval (0,1).In the ANOVA model, the three sets of parameters $\boldsymbol{\phi_i}$, $\boldsymbol{\delta_i}$ and $\boldsymbol{\gamma_{it}}$ are all random effects, follow **NCAR** prior distributions. The models have been implemented as the ST.CARlinear model in the package [29]. The argument model="linear" for linear, model="anova" for ANOVA and model="ar" was used for one order autoregressive and the autoregressive model of order two, AR=2 argument was used in this study for autoregressive model and "CarBayesST" package chooses this model in the bmstdr package under the Bcartime function. The “ggplot2” package in RStudio was used to visualize the variables. The shape files were also read using the “readOGR” function. The R programming language and the "Bcartime" package were used for geographical analysis and model fitting (Sahu, 2021). To select the appropriate model, Bayesian

model selection criteria such as the Log Marginal Predictive Likelihood (LMPL), Deviance Information Criterion (DIC) [30] and Watanabe-Akaike Information Criterion (WAIC) [31] were used in this study along with RMSE and MAE were used for evaluating the accuracy of the best model.

Ethical Statements: Not applicable.

Results

Exploratory Data Analysis (EDA) is a crucial step in the data analysis process where the main goal is to summarize the main characteristics of a dataset, often with the help of graphical representations. It is assumed that $\boldsymbol{Y_{it}}$ denotes annual literacy rate of $\boldsymbol{i^{th}}$ district and $\boldsymbol{t^{th}}$ year. Here, $\boldsymbol{i}$ denotes for district and $\boldsymbol{i = 1, 2, \ldots, 64}$ districts in Bangladesh and $\mathbf{t = 1, 2, \ldots, 21}$ for 21 years from 2001 to 2021.

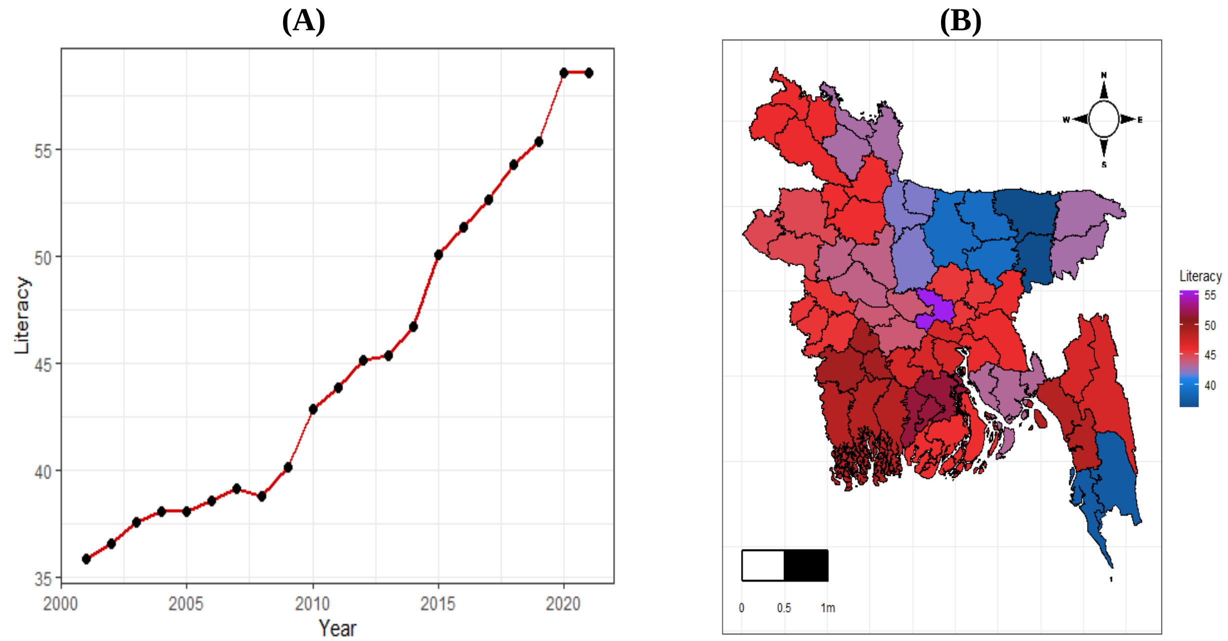

Figure 1: Time series plot (left panel), Spatial distribution of Literacy rate (right panel) in Bangladesh

Time series plot [Figure 1 (left panel)] of literacy rates in Bangladesh's 64 districts reveals a consistent increase in literacy over the past three decades. In 2020, Dhaka district had the highest

literacy rate due to its role as the education hub. Conversely, Habiganj showed the lowest literacy rate between 2001 and 2021, with Mymensingh, Kishoreganj, Sherpur, and Cox's Bazar also lagging in literacy levels, particularly in the southeast region [Figure 1 (right panel)].

~~To explore the relationship between literacy rate and the other social index, the authors used a correlation matrix presented in Figure 2~~. The analysis of Bangladesh's literacy rate and related variables reveals several key findings. There is a positive association between literacy rate and variables such as health index (0.77), income index (0.85), expected years of schooling (0.94), and population density (0.19). Wealth and health positively influence literacy rates. However, there is only a weak positive correlation between population density and literacy rate, mainly seen in urban areas. Conversely, a high dependency ratio (-0.77) is strongly negatively correlated with the literacy rate, indicating that as dependency increases, literacy tends to decrease. The distance from Dhaka to different districts shows no significant correlation with the literacy rate in the country.

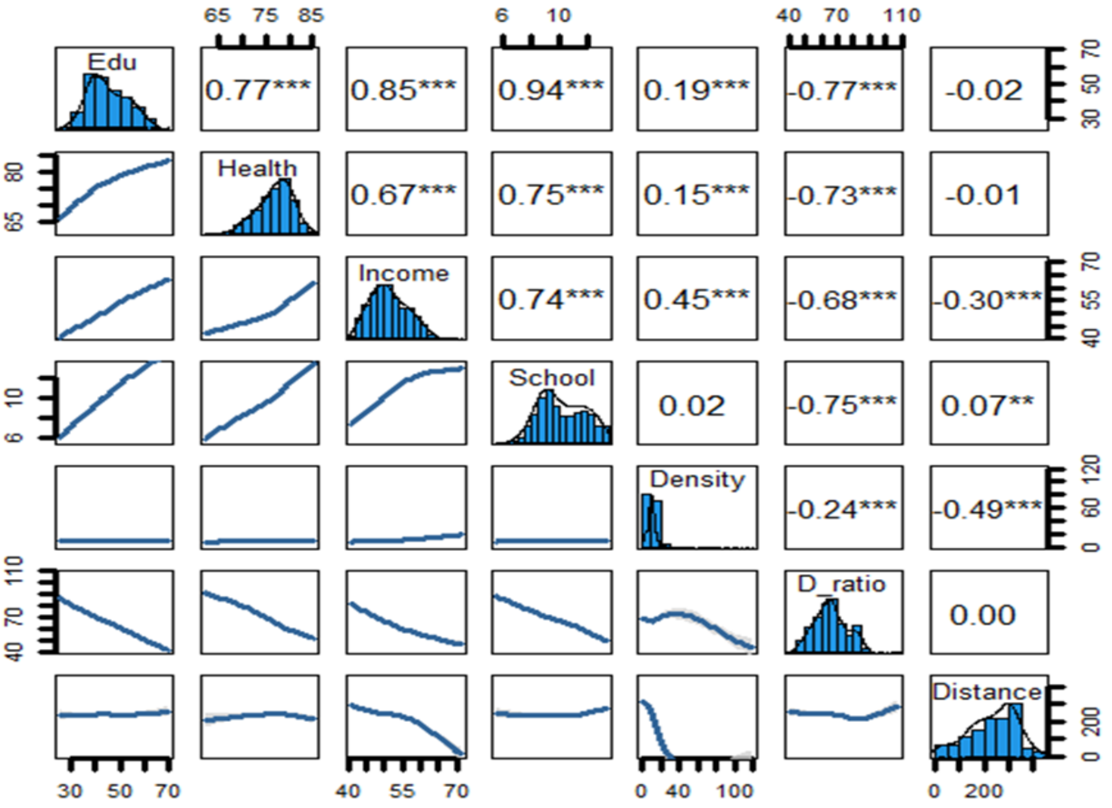

Figure 2: Correlation matrix of the selected variables, *** presents p-value <0.001, ** presents p-value <0.05, * presents p-value<0.1

Additionally shown in Figure 3 is the type of important covariates by spatial domain. The plot illustrates significant regional disparities in Bangladesh. Dhaka and the southeastern, and northeastern regions exhibit better health outcomes, while the coastline regions have fewer health facilities. Most districts have below-standard living conditions, with high-income indices mainly seen in Dhaka and a portion of the northwest. Barishal, Jhalokati, and Pirojpur show higher predicted years of schooling attendance. Dependency ratios are lower in Dhaka and negatively correlated with literacy. The population density is highest in Dhaka, contrasting with the mountainous region's lower density, and the northeastern districts are distant from the capital.

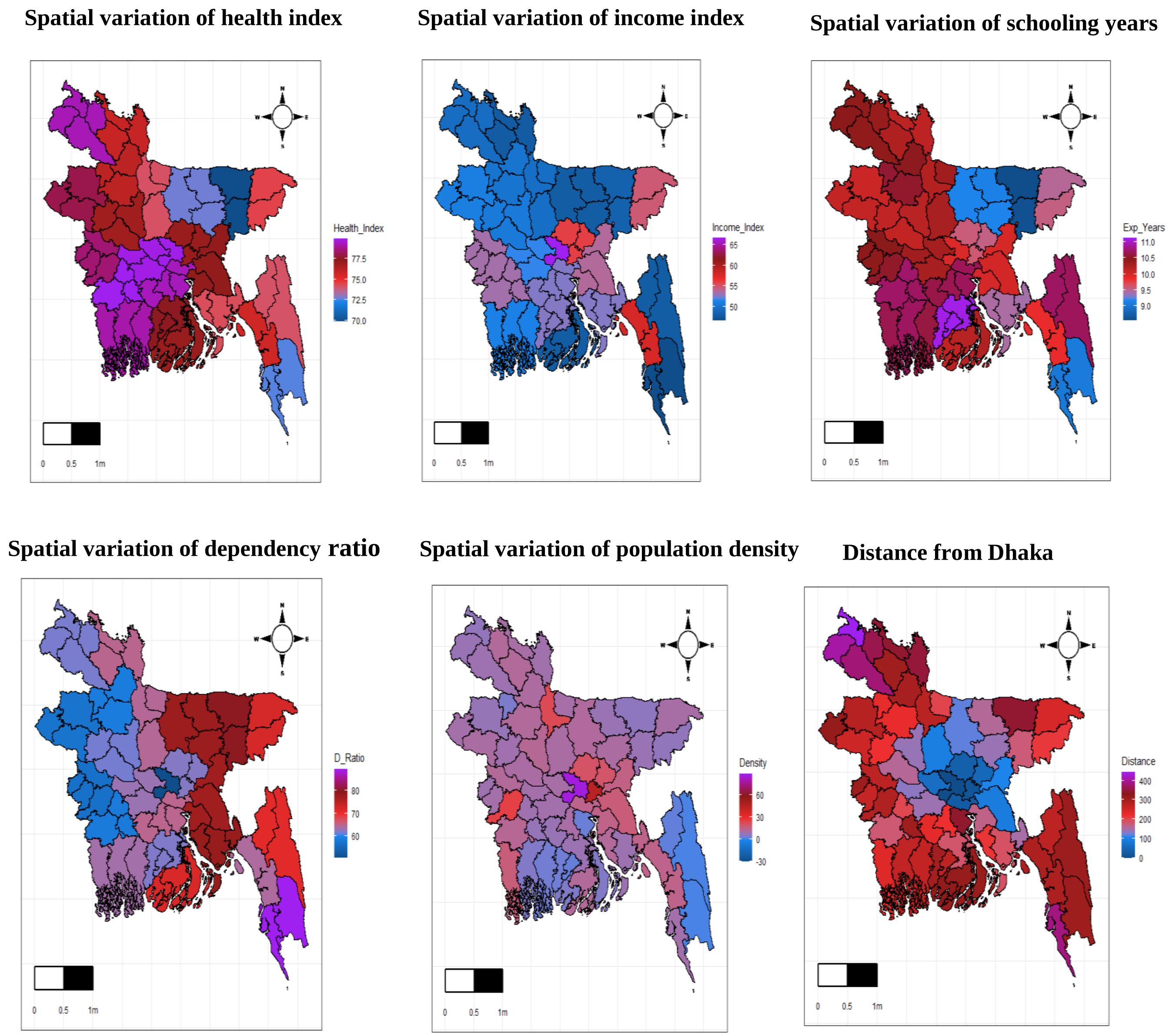

Figure 3: District-wise variation of significant covariates

To examine autocorrelation with Global Moran's I let set the hypothesis as,

$\mathbf{H_0}$ = There is no spatial dependence between neighboring districts due to educational developement.

$\mathbf{H_1}$ = There is spatial dependence between neighboring districts due to educational developement.

Table 1: Moran's I and Geary's C Indicators for literacy Rate

Summary Statistics	Global Moran's I	Geary's C
**Statistic**	0.486	0.542
**Expectation**	-0.0158	1.000
**Variance**	0.0067	0.0083
**Standard Deviate**	6.141	5.0174
**P -value**	4.103×10^{-10}	2.619×10^{-7}

The Table 1 presents the Global Moran's I and Geary's C statistics for literacy rate as development indicator. Moran I's yielded a score of 0.486, indicating a considerable spatial autocorrelation between regional domains. The null hypothesis should be rejected, as indicated by the obtained p-value of 4.103×10^{-10}, which is less than 0.05. Moran' I demonstrate the p-value of 5×10^{-5}, at a 5% threshold of significance using MC simulation of the 19999 global Moran. The null hypothesis should be disregarded in both scenarios.

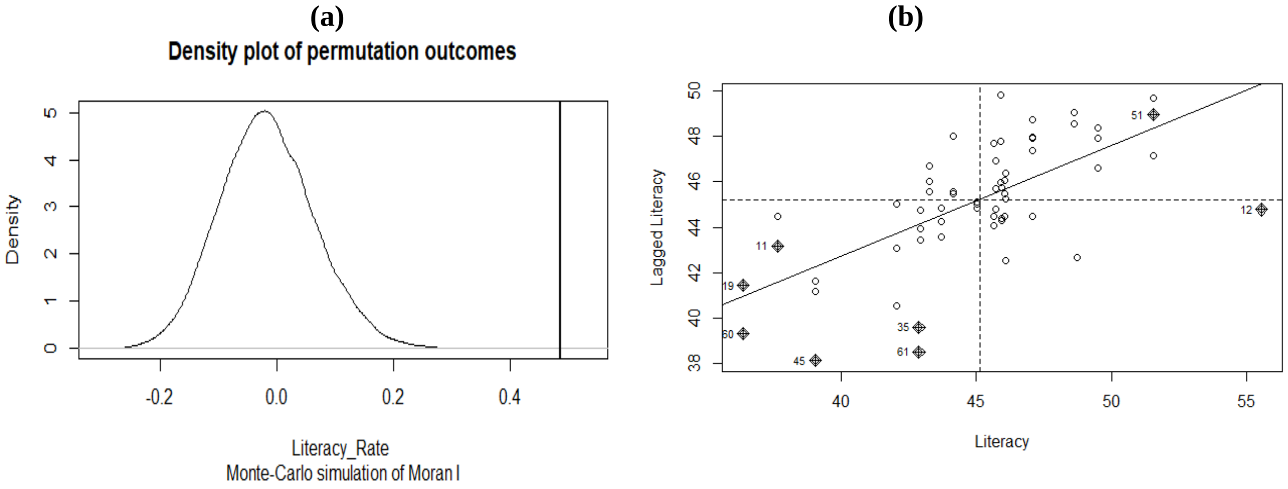

Figure 4: (a) Density plot of Global Moran's I, (b) Graphical representation of spatial autocorrelation

A density plot, Figure 4(a) of the Monte Carlo permutation results shows yet another example of how likely the observed test statistic is. The plot shows permutation outcomes on a curve and the Moran’s I value–the latter being so far to the right of the curve, at a value of 0.486, indicates positive spatial autocorrelation is exit. A positive autocorrelation between districts is also shown by the derived Geary’s C statistic value of 0.542, which is inside the [0,1) interval. The above two spatial autocorrelation analysis methods reveal the presence of clusters between districts' literacy percentages. To visualize the spatial autocorrelation in the dataset we constructed a “Moran scatter plot” which displays the spatial data against its spatially lagged values in Figure 4(b). According to the global evaluation of the dataset (positive Moran’s *I* value), there is spatial autocorrelation and it must be reduced in the MCMC setting in modeling section.

Similar to the global analysis (above), local Moran's I base the test statistic on the spatial weights of the items. While the traditional Moran's I statistic offers a valuable measure of global spatial autocorrelation in illiteracy rates, it falls short of capturing local spatial patterns. Figure 5 demonstrates that such districts have low educational development along with the low neighboring districts.

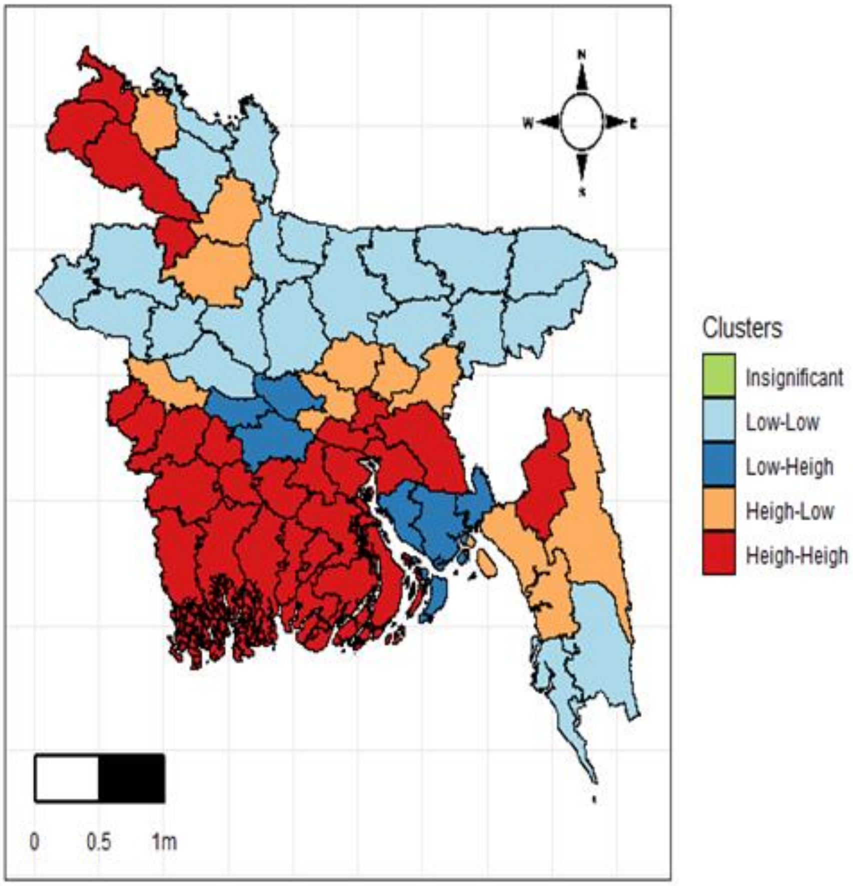

Figure 5: Spatial Clustering (Local Moran's I) of Literacy Rate

The following districts are part of the Low-Height spatial cluster and meet the criteria for statistical significance: Noakhali, Manikganj, Faridpur, Feni, Lakshmipur, and Rajbari. It would seem that these districts have a low literacy rate, while it would also seem that the districts next to them have a high literacy rate. The brown color code in the graph indicates that 10 districts give the impression of having a high literacy rate. In the graph another 27 districts are in the High-High Cluster category, indicating that they have neighboring districts containing a high educational development. So, a clear concept of clustering is found in the above graphical representation.

Exploratory spatial data analysis (ESDA) has difficulties in capturing the temporal dynamics of geographical features, even though it can be used to depict and summarize complicated spatial patterns [32]. To capture temporal dynamics along with spatial effect, Bayesian spatio-temporal

modeling is an excellent approach. It is preferable to start with an Independent Error Bayesian model before beginning modeling with the spatial and temporal domains so that the improvement when the spatial and temporal effects are introduced into the model can be appreciated. The linear predictor $x_{it}'\beta$ can be specified in the model as,

Health Index + Income Index + Expected Years Schooling + Population Density + Dependency Ratio + Distance.

Here population density is considered per 100 kilometers and the distance from Dhaka is measured in kilometer. After 20,000 burn-in iterations, all of the models were run for 120,000 iterations. To reduce autocorrelation in the MCMC setting, the data were kept following a thinning of 10 iterations. The fitted bmstdr model yields the following estimate including the confidence interval for all five models in Table 2. This table contains different spatial and temporal indicator values for the four spatio-temporal models, described above. The analysis reveals the significant impact of expected years of schooling on educational development (literacy rate), across all models. Additionally, health and income indices exhibit considerable influence on literacy percentages. However, the variable "distance" exerts a relatively minor effect on literacy rates, as indicated by the credible intervals in Table 2. The dependency ratio is also insignificant which matches with the pair-wise scatter plot. Thus, all values provide insights into the relationships between the predictor variables and the response variable. The estimate of the error variance ν^2 is 3.919 is higher for Independent Error Bayesian GLM as spatial and temporal variance is not considered yet. In the case of spatio-temporal linear models, ${\tau^2}_{int}$= 0.161 signifies lower variance for intercept than for slope, ${\tau^2}_{slo}$=0.777. An estimated spatial correlation parameter, ρ=0.693, suggests strong evidence of spatial correlation, further supported by ρ_{int}=0.586, indicating higher auto-regression for the intercept compared to ρ_{slo}=0.412. The ANOVA model in Table 2 displays a spatial correlation value of ρ_S is 0.576, indicating significant spatial influence and is higher than the value of $\rho_T = 0.419$ which indicates that spatial variation of literacy rate is strongly dependent on along with temporal effect.

Table 2: Estimated parameters and credible intervals for all proposed models

	Independent Error Bayesian GLM	Bayesian Spatio-Temporal Model			
		**Linear**	**ANOVA**	**AR (1)**	**AR (2)**
	**Estimate**	**Estimate**	**Estimate**	**Estimate**	**Estimate**
	**CI**	**CI**	**CI**	**CI**	**CI**
**Intercept**	-21.369	-18.810	-16.283	-16.247	-16.164
	(-25.233, -17.468)	(-22.716, -14.928)	(-20.131, -12.395)	(-20.054, -12.392)	(-19.971, -12.317)
**Health Index**	0.106	0.095	0.049	0.049	0.047
	(0.061, 0.150)	(0.050, 0.139)	(0.006, 0.092)	(0.006, 0.091)	(0.005, 0.090)
**Income Index**	0.500	0.487	0.544	0.543	0.543
	(0.460, 0.541)	(0.446, 0.528)	(0.503, 0.585)	(0.502, 0.583)	(0.502, 0.584)
**Years of Schooling**	3.200	3.189	3.053	3.061	3.062
	(3.063, 3.342)	(3.051, 3.330)	(2.917, 3.188)	(2.926, 3.192)	(2.932, 3.196)
**Population Density**	0.027	0.024	0.021	0.021	0.021
	(0.015, 0.040)	(0.011, 0.036)	(0.008, 0.033)	(0.008, 0.033)	(0.008, 0.034)
**Dependency Ratio**	-0.017	-0.030	-0.035	-0.035	-0.035
	(-0.034, -0.001)	(-0.047, -0.013)	(-0.052, -0.019)	(-0.051, -0.019)	(-0.051, -0.019)
**Distance**	0.004	0.004	0.003	0.003	0.003
	(0.003, 0.005)	(0.003, 0.005)	(0.002, 0.004)	(0.002, 0.004)	(0.002, 0.004)
$\tau^2{}_S$	-	**-1.353**	**0.159**	-	-
		(-1.724, -0.984)	(0.084, 0.285)		
$\tau^2{}_T$	-	**0.161**	**0.933**	0.290	**0.281**
		(0.085, 0.290)	(0.500, 1.690)	(0.139, 0.496)	(0.133, 0.512)
ν^2	3.919	**3.721**	**3.114**	2.988	2.983
	(3.627, 4.233)	(3.442, 4.013)	(2.890, 3.361)	(2.747, 3.244)	(2.754, 3.248)
ρ_S	-	-	**0.576**	**0.994**	**0.994**
			(0.170, 0.937)	(0.986,0.998)	(0.987, 0.998)
ρ_T	-	-	**0.419**	**0.347**	-
			(0.036, 0.855)	(0.052, 0.638)	
α	-	0.777	-	-	-
		(0.189, 2.181)			
$\tau^2{}_{int}$	-	-	-	-	-
$\tau^2{}_{slo}$	-	-	-	-	-
ρ_{int}	-	0.586	-	-	-
		(0.169, 0.939)			
ρ_{slo}	-	0.412	-	-	-
		(0.016, 0.925)			
$\rho1_T$	-	-	-	-	0.247
					(-0.131, 0.625)
$\rho2_T$	-	-	-	-	-0.082
					(-0.416, 0.277)

Note: CI: Credible interval

The values of $\tau^2{}_S$ and $\tau^2{}_T$ for the ANOVA model in the Table 2 are non-zero, which supports the necessity of taking the spatial and temporal domain into account in the model as well as the

existence of geographical and temporal variation in the literacy percentage. According to estimates of the AR (1) model, the temporal auto-correlation parameter $\rho_T = 0.347$ is roughly three times smaller than the spatial correlation value $\rho_S = 0.994$. which indicates a high spatial correlation exists in the model. Both the AR parameters of the AR (2) model show a negative lagged correlation which can be interpreted from the confidence interval of the estimated value. Thus, from above it is clear that that are both spatial and temporal effects of variables on literacy percentage in Bangladesh. Ultimately, the results in Table 2 underscore the joint influence of both spatial and temporal factors on literacy percentages in Bangladesh.

Table 3: Performance measures of the selected models

**Models**	**Model Selection Criteria**						**Model validation Criteria**			
	**DIC**	**p. d**	**WAIC**	**p. w**	**LMPL**	**loglikelihood**	**RMSE**	**MAE**	**CRPS**	**CVG**
**IEBGLM**	5659.89	7.963	5661.44	9.365	-2830.72	-2822.000	1.946	1.532	1.179	94.029
**Linear**	5619.97	36.69	5622.25	37.89	-2811.23	-2773.340	1.861	1.465	1.036	94.776
**AR (1)**	5375.25	88.25	5379.65	87.05	-2690.53	-2599.371	1.770	1.388	0.936	94.029
**AR (2)**	5377.82	85.49	5382.38	84.78	-2691.93	-2602.584	1.773	1.391	1.059	94.029
**ANOVA**	5388.86	45.73	5391.64	46.75	-2695.91	-2648.663	1.735	1.395	1.093	94.029

It is extremely important to choose a fitted model to find the best model that best fits the data. Table 3 combines all performance measure of the selected models to choose the best one based on model selection criteria and validation criteria and Figure 5 also presents model comparison plots of criteria which is performed using the MCMC sample. Table 3 and Figure 5 reveal that the AR (1) model outperforms others with the lowest WAIC and DIC values. Additionally, AR (1) exhibits the highest log marginal predictive likelihood (LMPL) and log likelihood values compared to other spatiotemporal models. Model validation criteria further support AR (1) with lower RMSE, MAE, and CRPS. Notably, the spatio-temporal linear model demonstrates the highest convergence rate. Consequently, AR (1) emerges as the best-fitting model for data analysis.

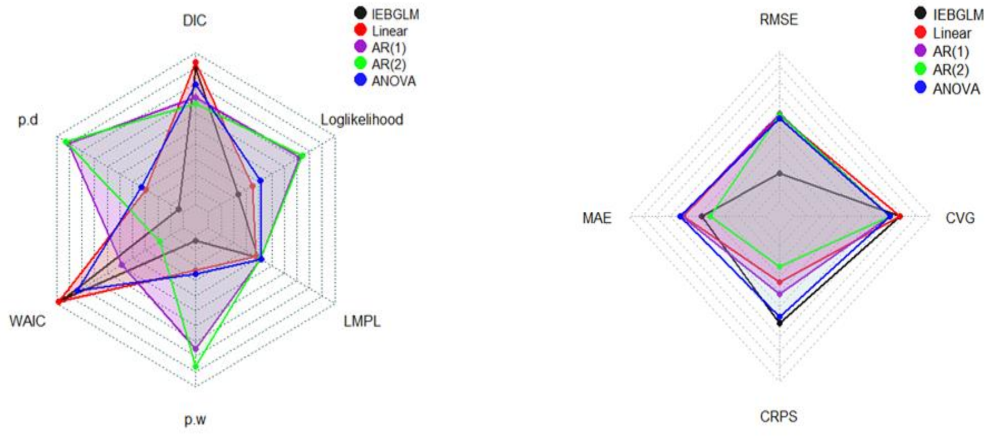

Figure 5: Comparison plot for Performance measures of the selected models

For each observed response, the MCMC iteration generates fitted values, and from fitted values residuals are computed for fitted plot construction. The spatial residuals and standard deviation of the residuals are then calculated for each spatial domain (district) based on the range of the temporal domain. Here, 10000 samples from the MCMC are used to calculate the residuals and their standard deviation.

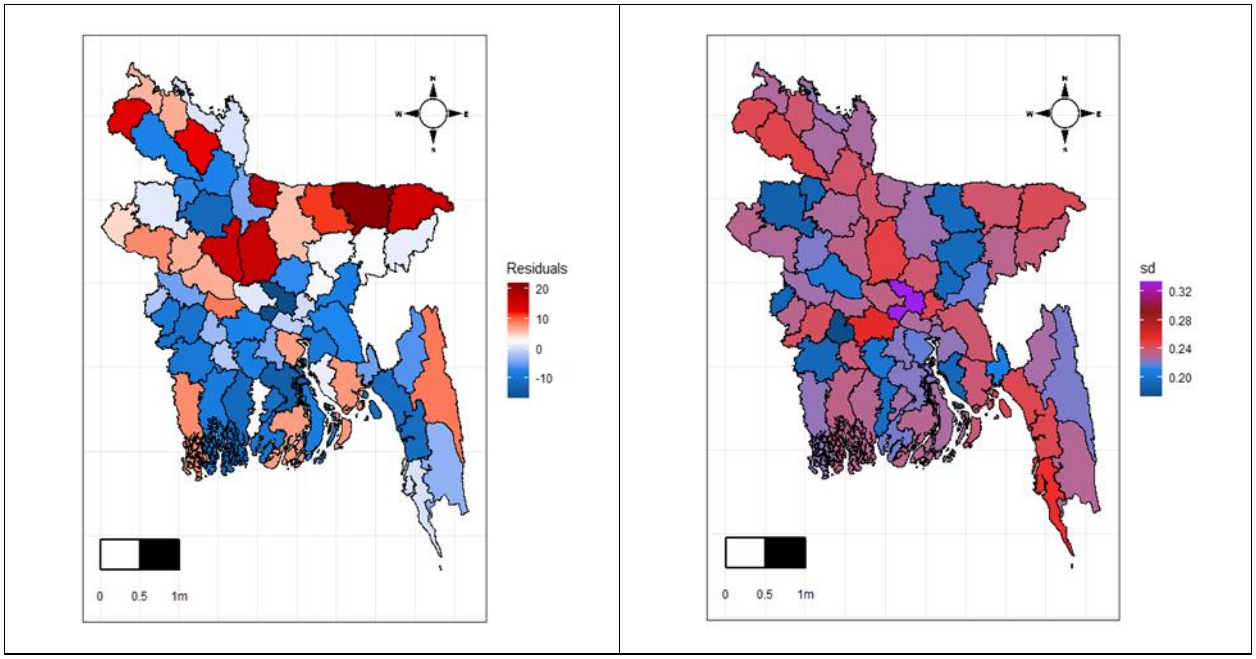

Figure 6: District-level, Spatially Aggregated Residuals (left panel) and Residual Standard Error (right panel)

Figure 7 displays a residual plot for each district as well as a plot for the residual's standard error. It is evident from the standard error plot that the residual values do not vary excessively. The plots in Figure 6 do not reveal any noteworthy spatial patterns that call for further research. Instead, the residuals from the spatio-temporal Autoregressive (AR(1)) model are represented by the spatially aggregated residual and the standard deviation of the residuals.

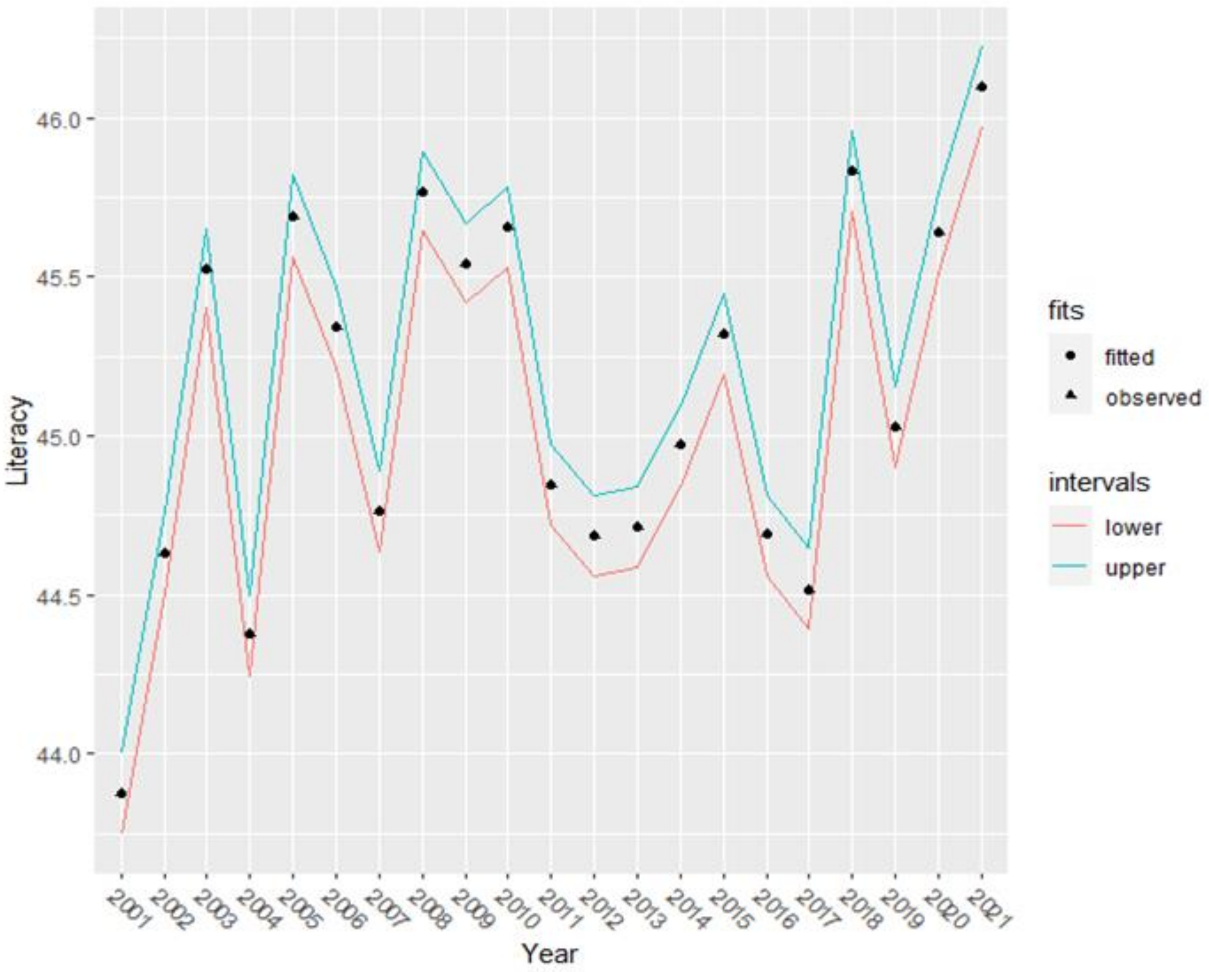

Figure 7: Observed and Fitted Time-series plot for Literacy Rate with 95% Confidence Intervals

Over the entire period (21 years), the fitted values appear to be fairly balanced. The plot shows neither a major exaggeration nor an underestimate. Indicating a good accuracy of the AR (1) model for the spatial-temporal framework, the lower and upper lines are also pretty near to the fitted values [Figure 7].

Discussion

Development disparities among regions are a prevalent occurrence, observed in both developed and developing nations, with more pronounced variations in the latter, such as Bangladesh, where progress unevenly advances across different geographical areas. This article attempts to find out the spatial and temporal variations in educational development (Literacy) at regional levels in the country. The findings of this study depict that in Bangladesh at the district level, the literacy rate has been growing over the years, but there exists inequity in its rates among districts. A previous study also observed that the literacy rate has increased significantly in Bangladesh to achieve SDG-4 [6]. A vital prerequisite for accelerating the achievement of other Sustainable Development Goals is guaranteeing all students have access to high-quality education [33] and a well-diversified education system [34]. Numerous studies have highlighted the strong connection between socioeconomic status and literacy rates in Bangladesh. Lower-income families often struggle to afford education-related expenses, leading to reduced access to quality education [17,18]. Like, Khan and Islam examined the relationship between poverty and educational attainment [35] this study has highlighted spatial variations in education facilities by identifying developed and lagging regions in the country.

In our study, we observe a noteworthy influence of the income index on literacy rates, affirming that higher family income is associated with elevated levels of literacy. This underscores the reciprocal relationship between income and literacy, indicating that while illiteracy may not be the cause of poverty, poverty indeed contributes to lower literacy levels. The health and nutritional status of individuals, particularly children, can affect cognitive development and, subsequently, their ability to learn and become literate [19]. Our research confirms a discernible impact of the health index on the overall literacy rate, highlighting their interconnected relationship. Historically, global literacy rates have significantly increased over the past two centuries, primarily due to higher enrollment in primary education and substantial growth in secondary and tertiary education [20]. Today, average years of schooling are substantially higher compared to a century ago and our study represents the high influence of expected years of schooling on literacy rate. Again, Urban areas tend to have better educational facilities and resources, leading to higher literacy rates compared to rural regions and research often focuses on this [22]. Academic

performance is below the national average in several areas of Bangladesh that are marked by remoteness and ecological problems [23]. Geographic disparities in terms of access to schools, transportation, and infrastructure often result in lower literacy rates in remote or underdeveloped areas [36]. Inequality in service delivery across adjacent town neighborhoods is discovered as a result, and the overall spatial distribution of the schools exhibits a clustered pattern [11]. Prior analysis [37] revealed a degree of spatial coherence in the distribution of literacy rates and the presence of educational institutions in Bangladesh. Our study reaffirms the presence of spatial consistency in the distribution of literacy rates and disparities in the availability of educational facilities in the country.

Conclusion

This study revolves around a comprehensive assessment of the interplay between spatial and temporal dimensions impacting educational development within Bangladesh. Additionally, we were keen to identify the multifaceted factors influencing educational development. In this study the spatial-temporal model has used to fulfill the objectives of the study in a Bayesian framework. The findings of our analysis indicate the presence of both spatial and temporal effects on educational development, specifically the literacy rate in Bangladesh. Hence, from the array of Bayesian Hierarchical Spatio-Temporal models explored, the Auto Regressive model of order one (AR (1)) emerges as the optimal choice for modeling our educational development index data, effectively capturing both spatial and temporal intricacies and this model has a satisfactory level of convergence on MCMC. The study reveals the significant impact of expected years of schooling on educational development (literacy rate). Additionally, health and income indices exhibit considerable influence on literacy percentages. However, the variable "distance" exerts a relatively minor effect on literacy rates and the dependency ratio hurts it. The results of this paper suggest that although the government has unique strategies for the districts outside of Dhaka, they are insufficient. This would enable them to develop the required policies and put development initiatives into practice. This study, by considering both spatial and temporal dimensions, provides policymakers with a holistic grasp of the complex aspects of regional development disparities. In conclusion, the findings point to the necessity of concentrating differently on community-level programs aimed at boosting access to health care and educational opportunities, particularly in

Bangladesh's underprivileged districts. Policymakers should prioritize given initiation which can be recommended from the outcome of our research. Furthermore, the authors are hopeful that the findings will be motivating for the researchers who want to examine how development indices are affected by time and region, which they may use to get a concise assessment of the features of various districts in Bangladesh in terms of development indicators.

Funding: This research received no specific grant from funding agencies in the public, commercial, or not-for-profit sectors.

Data Availability Statement: All Data are available in the website of Global Data Lab (https://www.globaldatalab.org) and Bangladesh Bureau of Statistics (https://bbs.gov.bd/).

Author Contributions: Afroza Sultana: Conceptualization, Data curation, Formal analysis, Methodology, Software, Visualization, Investigation, Writing – original draft. Md. Akher Ali: Data curation, Visualization, Writing – original draft. Md. Sifat Ar Salan: Conceptualization, Formal analysis, Methodology, Software, Supervision, Investigation, Writing – original draft & editing. Mohammad Alamgir Kabir: Conceptualization, Methodology, Supervision, Writing – original draft & editing, Md. Moyazzem Hossain: Conceptualization, Methodology, Supervision, Writing – original draft & editing.

Reference

1. Gong K. Sustainable development and digitization. Engineering Education Review. 2024;2: 1–6. doi:10.54844/eer.2023.0490
2. McCowan T. Education as a human right: Principles for a universal entitlement to learning. Bloomsbury Publishing; 2013.
3. Andorno R. International Biolaw and Shared Ethical Principles. Caporale C, Pavone IR, editors. New York, NY : Routledge, 2017.: Routledge; 2018. doi:10.4324/9781315589312
4. Perveen R. A case study in progress from the KIX-EAP learning cycle “Equitable Access to Education with Geospatial Data” held in collaboration with IIEP. UNESCO. Bangladesh; 2021.
5. BBS. Statistical Year book of Bangladesh. In: Bangladesh Bureau of Statistics [Internet]. 2021 [cited 17 Oct 2023]. Available: http://www.bbs.gov.bd

6. Akther Wahida. Bangladesh’s Journey towards SDG 4: A Review of Progresses. International Journal of Multidisciplinary Informative Research and Review. 2022;2: 43–51.

7. Islam MR and MSHossain. Mathematical modeling of age specific adult literacy rates in Bangladesh. Advances in Life Sciences. 2014;4: 106–113.

8. Khan AM and II. Education and development in Bangladesh: A study from spatial perspective. Journal of Bangladesh Institute of Planners. 2010;3: 1–16.

9. Xiang L, Stillwell J, Burns L, Heppenstall A, Norman P. A geodemographic classification of sub-districts to identify education inequality in Central Beijing. Comput Environ Urban Syst. 2018;70: 59–70. doi:10.1016/j.compenvurbsys.2018.02.002

10. Lobban G. The Use of GIS to Manage Location Planning of Secondary Schools (A Case Study in Clarendon, Jamaica). Chicago State University. 2017.

11. Bulti DT, Bedada TB, Diriba LG. Analyzing spatial distribution and accessibility of primary schools in Bishoftu Town, Ethiopia. Spatial Information Research. 2019;27: 227–236. doi:10.1007/s41324-018-0227-6

12. Tobler WR. A Computer Movie Simulating Urban Growth in the Detroit Region. Econ Geogr. 1970;46: 234. doi:10.2307/143141

13. Smith MJ De, Goodchild Michael F., Paul Longley. Geospatial analysis: a comprehensive guide to principles, techniques and software tools. Troubador publishing ltd. 2007.

14. Sahu SK. Hierarchical Bayesian Models for Space–Time Air Pollution Data. 2012. pp. 477–495. doi:10.1016/B978-0-444-53858-1.00016-8

15. Rachmawati RN, Pusponegoro NH. Spatio-Temporal Poverty Analysis with INLA in Hierarchical Bayes Ecological Regression. Procedia Comput Sci. 2018;135: 323–330. doi:10.1016/j.procs.2018.08.180

16. GDL. Global Data Lab. 2021 [cited 17 Oct 2023]. Available: https://globaldatalab.org//ddw/

17. Ahmed S. Addressing the Challenge of Income Inequality in Bangladesh (No. 6). PRI Working Paper Series. 2014.

18. Street D. Social illiteracies. London and New York: Longman. 1995; 9–15.

19. Sarma H, Khan JR, Asaduzzaman M, Uddin F, Tarannum S, Hasan MdM, et al. Factors Influencing the Prevalence of Stunting Among Children Aged Below Five Years in Bangladesh. Food Nutr Bull. 2017;38: 291–301. doi:10.1177/0379572117710103

20. Vanderslott S, Dattani S, Spooner F, Roser M. Vaccination. Our World in Data. 2013 [cited 17 Oct 2023]. Available: https://ourworldindata.org/vaccination

21. Mazumdar K. Socio-economic factors determining adult literacy in developing countries. Int J Soc Econ. 2005;32: 98–120. doi:10.1108/03068290510575667

22. Kabir AH, Akter F. Parental Involvement in the Secondary Schools in Bangladesh: Challenges and a Way Forward. International Journal of Whole Schooling. 2014;10: 1–18.

23. Ahmed M, Rahaman KP. Skills development in Bangladesh: Enhancing the youth skills profile. Dhaka, Bangladesh: Campaign for Popular Education (CAMPE) Bangladesh; 2013.

24. Geary RC. The Contiguity Ratio and Statistical Mapping. The Incorporated Statistician. 1954;5: 115. doi:10.2307/2986645

25. Chen Y. New Approaches for Calculating Moran's Index of Spatial Autocorrelation. PLoS One. 2013;8: e68336. doi:10.1371/journal.pone.0068336

26. Chen Y. Spatial Autocorrelation Approaches to Testing Residuals from Least Squares Regression. PLoS One. 2016;11: e0146865. doi:10.1371/journal.pone.0146865

27. Shen C, Li C, Si Y. Spatio-temporal autocorrelation measures for nonstationary series: A new temporally detrended spatio-temporal Moran's index. Phys Lett A. 2016;380: 106–116. doi:10.1016/j.physleta.2015.09.039

28. Knorr-Held L, Raßer G. Bayesian Detection of Clusters and Discontinuities in Disease Maps. Biometrics. 2000;56: 13–21. doi:10.1111/j.0006-341X.2000.00013.x

29. Sahu SK. bmstdr: Bayesian Modeling of Spatio-Temporal Data with R. Technical Report, submitted. 2021 [cited 17 Oct 2023]. Available: https://www.soton.ac.uk/sks/bmbook/bmstdr-vignette.html

30. Spiegelhalter DJ, Best NG, Carlin BP, Van Der Linde A. Bayesian Measures of Model Complexity and Fit. J R Stat Soc Series B Stat Methodol. 2002;64: 583–639. doi:10.1111/1467-9868.00353

31. Watanabe S. Asymptotic equivalence of Bayes cross validation and widely applicable information criterion in singular learning theory. Journal of machine learning research. 2010;11: 3571–3595.

32. Wang Z, Lam NSN. Extending Getis–Ord Statistics to Account for Local Space–Time Autocorrelation in Spatial Panel Data. The Professional Geographer. 2020;72: 411–420. doi:10.1080/00330124.2019.1709215

33. Tang Q. Goal 4—Education in the Post-2015 Sustainable Development Agenda. Apr 2015 [cited 17 Oct 2023]. Available: https://www.un.org/en/chronicle/article/goal-4-education-post-2015-sustainable-development-agenda

34. Alam GM. The role of technical and vocational education in the national development of Bangladesh. International Journal of Work-Integrated Learning. 2008;9: 25–44.

35. Khan A, Islam I. Education and Development in Bangladesh: A Study from Spatial Perspective. 2010.

36. Barman S, Farid F Al, Uddin N, Seddiqui H. A semantic knowledgebase representation of geospatial data in the context of Bangladesh. 2014 International Conference on Informatics, Electronics & Vision (ICIEV). IEEE; 2014. pp. 1–6. doi:10.1109/ICIEV.2014.7136005

37. Zahiduzzaman AKM, Mohammed Nahyan Quasem, Khan M, Rahman RM. Spatial data mining on literacy rates and educational establishments in Bangladesh. 2010 13th International Conference on Computer and Information Technology (ICCIT). IEEE; 2010. pp. 394–399. doi:10.1109/ICCITECHN.2010.5723890
